# Unique Alterations of an Ultraconserved Non-Coding Element in the 3′UTR of *ZIC2* in Holoprosencephaly

**DOI:** 10.1371/journal.pone.0039026

**Published:** 2012-07-31

**Authors:** Erich Roessler, Ping Hu, Sung-Kook Hong, Kshitij Srivastava, Blake Carrington, Raman Sood, Hanna Petrykowska, Laura Elnitski, Lucilene A. Ribeiro, Antonio Richieri-Costa, Benjamin Feldman, Ward F. Odenwald, Maximilian Muenke

**Affiliations:** 1 Medical Genetics Branch, National Human Genome Research Institute (NHGRI), National Institutes of Health, Bethesda, Maryland, United States of America; 2 Zebrafish Core Facility, Genetics and Molecular Biology Branch, National Human Genome Research Institute (NHGRI), National Institutes of Health, Bethesda, Maryland, United States of America; 3 Genome Technology Branch, National Human Genome Research Institute (NHGRI), National Institutes of Health, Bethesda, Maryland, United States of America; 4 Molecular Genetics Laboratory and Clinical Genetics Service, Hospital for Rehabilitation and Craniofacial Anomalies, USP, Bauru, Brazil; 5 Neural Cell-Fate Determinants Section, National Institute of Neurological Diseases and Stroke, National Institutes of Health, Bethesda, Maryland, United States of America; Oslo University Hospital, Norway

## Abstract

Coding region alterations of *ZIC2* are the second most common type of mutation in holoprosencephaly (HPE). Here we use several complementary bioinformatic approaches to identify ultraconserved cis-regulatory sequences potentially driving the expression of human *ZIC2*. We demonstrate that an 804 bp element in the 3′ untranslated region (3′UTR) is highly conserved across the evolutionary history of vertebrates from fish to humans. Furthermore, we show that while genetic variation of this element is unexpectedly common among holoprosencephaly subjects (6/528 or >1%), it is not present in control individuals. Two of six proband-unique variants are *de novo*, supporting their pathogenic involvement in HPE outcomes. These findings support a general recommendation that the identification and analysis of key ultraconserved elements should be incorporated into the genetic risk assessment of holoprosencephaly cases.

## Introduction

Holoprosencephaly (HPE, [MIM 236100]) is the most common congenital malformation of the forebrain in humans and involves varying degrees of deficient or incomplete separation of the cerebral hemispheres and deeper cortical structures along the CNS midline. HPE occurs in 1∶250 human embryos and is a major cause of both intra-uterine pregnancy loss and post-natal morbidity and mortality in affected cases [1]. Genetic factors contributing to HPE are numerous and the best understood genetic and/or environmental causes ultimately relate to defective formation and function of the axial midline [2] or positioning of a key ventral signaling center that patterns early forebrain structures [3]–[5].

Over a decade of clinical molecular research has identified at least four genes that should be routinely screened for mutations in HPE families: *SHH* [MIM 600725], *ZIC2* [MIM 603073], *SIX3* [MIM 603714] and *TGIF* [MIM 602630] [6]. Most diagnostic centers describe retrospective estimates of 20–25% of subjects with coding region alterations in these genes from the results of routine testing. Interestingly, virtually all of these mutations are found to be both heterozygous and unique. All commonly used diagnostic approaches consider only coding region segments in their analysis and clinical reports.

The mutational spectrum of human *ZIC2* is typical for a major HPE gene [7]. A substantial fraction of these mutations are predicted to eliminate the hypothetically translated protein's ability to function as a transcription factor and are therefore considered to be typical loss-of-function alleles [8]. The Zic family of transcription factors is a well-studied group that numbers at least five discrete members in higher vertebrates. This ancient gene family arose through multiple rounds of gene duplication, inversion and dispersal over at least three vertebrate chromosomes [9]–[10]. Both redundant and divergent functional roles have been established by systematic gene ablation in the mouse. Experimental murine alleles of *Zic2* are implicated in neurulation delay, neural tube defects and a spectrum of holoprosencephaly phenotypes [11]–[12].

In this report, we explore the likelihood that presumed regulatory regions in the vicinity of the *ZIC2* gene might be the target of genetic variation that could directly or indirectly influence the presence or manifestations of holoprosencephaly phenotypes. We noted at the outset that ultraconserved sequences are estimated to be quite common in the genome [13] and are particularly enriched in the neighborhood of developmental genes [14], such as *ZIC2* (reviewed in [15]). Furthermore, the precedent for HPE-associated enhancers had previously been advanced by the identification of a distal forebrain enhancer of the *SHH* gene that is regulated, in part, by a third HPE gene product, SIX3 [16]–[17]. Therefore, we now describe our general approach to the analysis of potential regulatory elements in the vicinity of human developmental genes and argue that the evolutionary constraints imposed by the pathophysiology of HPE promises a fruitful line of inquiry into ultraconserved gene regulatory networks responsible for major steps in forebrain specification.

## Materials and Methods

### Subjects and ethics statement

A total of 528 affected subjects were studied (436 from our NIH laboratory and 92 subjects from Brazil). All subjects provided written consent for research investigation of the genetic factors of holoprosencephaly presented to them in their native language. Commercially available anonymous controls were purchased from Sigma-Aldrich (4X 96 well plates: HRC1-4) or Coriell Institute for Medical Research (2X 96 well plates: COR1 and COR2); where indicated in Table 1, we used 76 ethnically matched anonymous Brazilian controls or a plate of 95 anonymous Asian individuals [HAPMAPPT02: Han Chinese, Japanese, both sexes]. Oversight of molecular analysis and results was provided by the IRB of the NHGRI, NIH and included the coded analysis of HPE samples consented independently in Brazil.

**Table 1:** *ZIC2* enhancer mutational screening and control results by TaqMan Assay.

Variant	Subject	# cases/total	MAF cases (%)	#controls/total	# controls/caucasian	# controls/ethnically matched	MAF controls (%)
c.1599*456G>A	**rs13542 dbSNP**	N.T.	-	-	-	-	33.0
c.1599*578T>A	LCL1349^a^	1/528	0.095	0/456	0/380	0/76 (Brazilian)	0
c.1599*587G>T	FB9622, LCL7282, LCL6386	3/528	0.28	5/380	5/380	-	0.66
c.1599*836C>T	Brz-2172^b^	1/528	0.095	0/372	0/288	0/75 (Brazilian)	0
c.1599*889T>C	AM6632	1/528	0.095	0/379	0/279	0/95 (Asian)	0
c.1599*899A>G	LCL301; LCL7897^c^	2/528	0.19	0/377	0/377	-	0
c.1599*954T>A	Brz-37^d^	1/528	0.095	0/452	0/367	0/76 (Brazilian)	0
c.1599*966A>G	LCL7828^e^	1/528	0.095	0/375	0/375	-	0

a *De novo*, parental testing confirms biological relatedness of parental DNA; ^b^ Variant allele *in cis* with a *ZIC2* c.1215dupC (p.Ser406Glnfs*91) based on co-amplification and subcloning; ^c^ Subject LCL7897 is the affected sibling of proband LCL301 (both are carriers of a SHH p.Cys24* mutation, see Table S1). ^d^ Described as *de novo* based on normal sequence of both parents (done in Brazil). ^e^ Proband also has novel mutations in *TGIF* (c.289A>G, p.Met97Val). SNPs are highlighted in bold.

### Amplification and mutation screening

For this study, the coding regions and immediate flanking intron-exon boundaries of the *SHH*, *ZIC2*, *SIX3* and *TGIF* genes were amplified by PCR and sequenced using an ABI 3100 genetic analyzer according to our CLIA lab procedures (available upon request). The reference sequences for these genes are NM_000193.2 (*SHH*), NM_007129.2 (*ZIC2*), NM_003244.2 (*TGIF*) and NM_005413.2 (*SIX3*). The summary of all genetic variants detected on an individual subject basis is described in Table S1. Any sequence variation determined in any of the genes tested was named using standard nomenclature rules http://www.hgvs.org/mutnomen/) and confirmed by on-line Name Checker using Mutalyzer (http://www.mutalyzer.nl/2.0). Comparison with public databases including 1000genome.org and dbSNP was performed to determine the uniqueness of the experimentally determined mutations.

For the putative enhancer element, synthetic oligonucleotide primers were designed and optimized to cover this non-coding sequence and immediate flanking sequences (amplicon 1: ZIC2enh_F [5′GTGTACATAGCGGACTCCTCCT3′] and ZIC2enh_R [5′GTCAATCCTCAGCTGCCTCTTC3′], product size 804 bp). PCR amplification was performed from 25 ng of genomic DNA template using the FastStart® Polymerase PCR Kit (Roche Applied Sciences, IN) on a 25 µL total reaction volume, under the following conditions: 1X (2.5 µl) of amplification buffer (10X containing 20 mM of MgCl_2_), 0.20 mM (0.5 µL) of dNTP mix (10 mM), 0.30 mM of each oligonucleotide primer, and 1U (0.2 µL) of FastStart® Polymerase (5 U/µL). Subsequently, PCR products were purified using QIAquick® 96 PCR purification kit (Qiagen, MD).

### DNA sequencing

Sequencing reactions were performed using the BigDye Terminator v3.1 chemistry and capillary electrophoresis was performed in an ABI 3730xl genetic analyzer (Applied Biosystems, CA) as recommended by the manufacturer. Chromatograms were aligned to the reference sequence (NM_007129.2) and analyzed using Sequencher version 4.9 (GeneCodes Corp, MI). TaqMan SNP genotyping assays were performed on LightCycler 480 II with dual-color hydrolysis assay program and the data were collected and analyzed utilizing Endpoint Genotyping analysis software (LightCycler 480 II, LightCycler 480 reagents and the software are available from Roche Applied Science).

**Figure 1:**
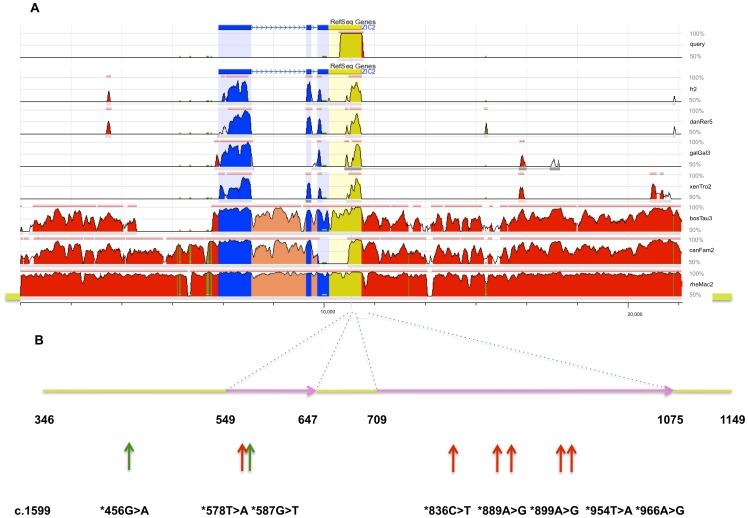
A Vista plot display of a multiple-species alignment of human *ZIC2*. (A) ECRbase view of the vertebrate *Zic2* regions (for these orthologs transcription from 5′ to 3′ is left to right) and where coding regions (blue), introns (orange), 3′ non-coding transcribed regions (yellow) and intergenic segments (red) are displayed. Vista plot peaks reflect the extent of homology (>50–100%) compared to the query sequence (human *ZIC2* 3′ UTR, 804 bp). (B) An enlargement of the two segments (pink: ECR#1_99 bp and ECR#2_367 bp) that retain conservation >50% between human and zebrafish in the 804 bp screened region (yellow). The positions of the polymorphic variations (green arrows) and unique variations (red arrows) are numbered from the last base of the stop codon (c.1599) of the human *ZIC2* reference sequence (NM_007129.2).

### Zebrafish husbandry and analysis

Zebrafish stocks and manipulations conformed to standard Animal Care and Use protocols used in the Zebrafish Core facility and Feldman lab, NHGRI, NIH. The Invitrogen Gateway entry vector pcr8®/GW/TOPO® was used to clone potential enhancer fragments that were then inserted into the ZED vector [18] (obtained under a Material Transfer Agreement) via a Gateway® LR Clonase II reaction following the manufacturer's instructions (Invitrogen, Carlsbad, CA). All microinjections were performed in one-cell stage embryos, containing 50 pg of transposase and 30 pg of purified DNA, following standard published protocols [19]. Dechorionated embryos for in situ hybridization and immunostaining were fixed in 1× PBS buffer containing 4% paraformaldehyde for 10 h at 4°C. Antisense digoxigenin-labeled *gfp* RNA probe was prepared from linearized template DNA using a DIG-RNA labeling kit (Roche). Whole-mount *in situ* hybridization was performed as described [20], except that post-hybridization washing was at 65°C. GFP antibody (Cell Signaling) was used at 1∶300 and biotinylated anti-rabbit IgG was used as secondary antibody (1∶500) (Vector Laboratories), following the manufacturer's instruction from R.T.U Vectastain kit. Whole-mount *in situ* hybridization patterns were observed with a Leica MZ16 dissecting microscope and photographed using with a Zeiss Axiocam HRc camera. Laser confocal microscopic images were obtained using a Zeiss LSM 501 META laser scanning microscope.

## Results

### Identification of potential regulatory regions and mutational analysis

As an initial step, we performed an EvoPrint [21] (http://evoprinter.ninds.nih.gov/evoprintprogramHD/evphd.html) of an arbitrarily selected 10 kilobase (kb) segment of human DNA encompassing the *ZIC2* gene as described by *ZIC2* (NM_007129.2) reference sequence annotation obtained from publicly available databases provided by NCBI (http://www.ncbi.nlm.nih.gov/) and the UCSC Genome Browser (http://www.genome.ucsc.edu/). As expected, we identified a strong signature of the C2H2 zinc-finger domain characteristic for this class of transcription factors that controls DNA binding ability (data not shown); however, we also noted an extremely conserved element in the non-coding 3′ UTR. Two independent analysis procedures using EvoPrinter and ECRbase (http://ecrbase.dcode.org/) identified a comparable region of conservation in the *ZIC2* 3′UTR that spanned nearly 800 bp (Figure 1A).

**Figure 2:**
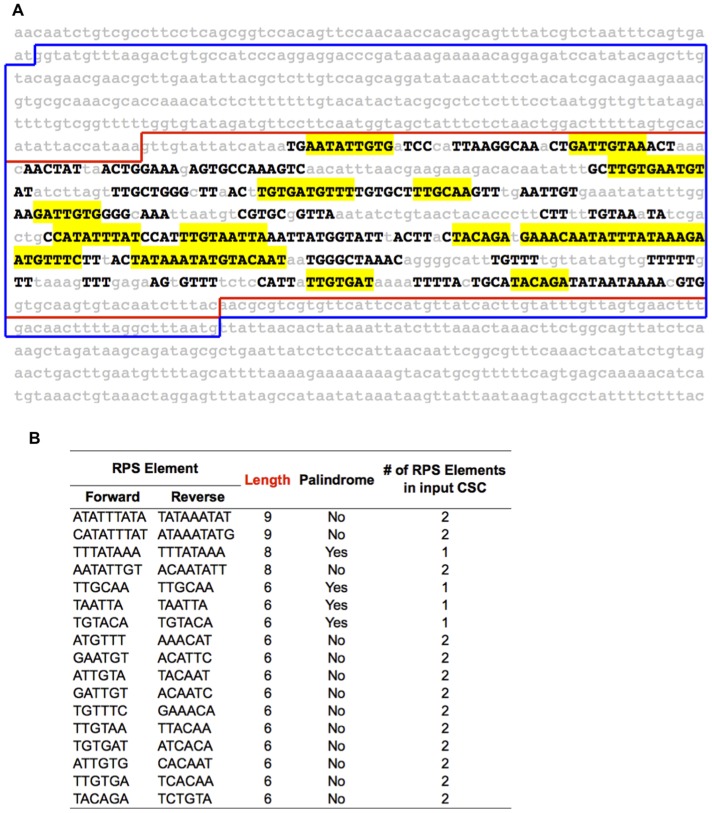
An EvoPrint view of a genomic segment of human DNA selected and then screened for mutations. (A) A multiple species comparison was performed where bases conserved in all but one of the test species, relaxed EvoPrint, appear as black uppercase letters (these alignments were among Human, Marmoset, Chimpanzee, Rhesus-Monkey, Horse, Platypus and Opossum) and are displayed in context with non-conserved bases (lower case, grey) spanning the entire 804 bp element shown in blue. (B) Analysis of the repetitive and palindromic structure of the EvoPrint using *cis*-Decoder identifies further substructure of its conserved sequence blocks (CSB). Distinct elements (>6 bp) are highlighted in yellow. These CSC analyzed and presented are contained within the element in common [>50% conservation between human and zebrafish, outlined in red] where most variants were detected.

Figure 1B shows an expanded view of the conserved region that includes all known genetic variants. Seven are experimentally detected variations (empirically polymorphic, green) or unique (red) are in one (ECR#1_99 bp) or the other (ECR#2_367 bp) of two DNA segments (pink) that are conserved between human and lower vertebrates. The eighth variant c.1599*456G>A (green) was also contained in the sequenced amplicon (but not the conserved ECRs above) and is a known SNP (rs13542, see Table 1) that is not common to multiple-species alignments (by EvoPrint, or PhyloP and PhastCon in UCSC [data not shown]; as well as ECRbase as shown in Figure 1A). According to UCSC Targetscan none of these 8 variants showed overlap with annotated microRNA binding sites (data not shown).

### Bioinformatic analysis of sequence motifs and position of variants

The internal predicted sub-structure of the enhancer element was determined using *cis*-Decoder, (http://cisdecoder.ninds.nih.gov/). As shown in Figure 2, our putative enhancer contains multiple short repeats that are typical of transcription factor docking sites. By using *cis*-Decoder we identify 17 distinct repeats of 6 bp or longer (highlighted yellow 2A, see data output file in 2B) covering 52% of the *ZIC2* element. This analysis revealed an internal conserved sequence structure of the 804 bp human sequence and identified multiple conserved sequence clusters (CSC) of repetitive or palindromic elements that are typical of neural enhancers in *Drosophila* [22] as well as higher vertebrates. Furthermore, a literature search identified precedent for functional enhancers in the 3′UTR of neural genes [23]. We also note that this sequence substructure is typical for neural enhancers and is composed of repeat and palindrome sequence elements. The evolutionary constraint on their conservation is tremendous and preserved over hundreds of millions of years of vertebrate divergence. As shown in Figure S1, a multiple-species alignment (UCSC PhastCon) of ECRbase elements ECR#1_99 bp and ECR#2_367bp shows that several of the unique variations from holoprosencephaly subjects [*889T>C and *954T>A] are in highly conserved sequence blocks as defined by PhastCon and *cis*-Decoder. Analysis of the predicted transcription factor binding sites of ECRbase alignments between human and mouse (Figure S2) and human and zebrafish (Figure S3) defines four regions within the 804 bp element that are docking sites for vertebrate transcription factors in TRANSFAC databases. Two of these regions are sites of mutation, namely the *889T>C and *954T>A changes are predicted to disrupt LIM and FOX class transcription factor binding sites, respectively.

### Transient transgenesis in zebrafish

In order to determine if the 804 bp putative enhancer was functional in zebrafish we cloned it into the ZED vector [18]. Embryos injected with negative control and experimental ZED vectors were analyzed and photographed at defined stages to document experimental (GFP) and internal control (RFP) expression (Figure S4). Examining transient transgenic embryos, we did not detect GFP fluorescent expression at any stage. Attempts to visualize lower *gfp* levels by *in situ* hybridization of transgene-injected embryos were confounded by artifactual signals from the vector where no corresponding GFP immunostaining was seen. This lack of measurable gfp is consistent with reports that under 50% of ultraconserved human elements yield tissue-specific expression in zebrafish [24], possibly indicating a lack of homologous zebrafish response apparatus, a need for additional flanking DNA from the human locus, or a combination of both factors.

## Discussion

As the costs of deep sequencing of clinical samples continues to come down and the extent of routine coverage increases from individual human coding segments towards whole genomes, it will become increasingly imperative that tools and techniques to predict or determine functional DNA from non-functional DNA keep apace. Here we have demonstrated that a combination of methods based on the assumption of evolutionary sequence conservation being a predictor of function is certainly one plausible approach. While it is also true that enhancers or related elements with similar regulatory potential need not be visibly conserved at the linear DNA alignment level [25], a deeper appreciation of enhancer substructure using *cis*-Decoder and related methodologies may well define recognizable commonalities among regulatory enhancers. One of the emerging principals from the analysis of *Drosophila* neural enhancers is that the repetitive and palindromic elements in a CSC are often preserved in kind and number, but not orientation or position [22]. This leads to a conclusion that it is the type of transcription factor binding sites, but not necessarily their position that may define functionality of enhancers.

Here we have demonstrated that using these types of tools we can identify selected putative cis-regulatory elements and test them for functionality in a convenient animal model using transient transgenesis in zebrafish (see also other examples, [25]–[26]). The method is estimated to be informative across distantly related vertebrate species in a substantial fraction of cases [27]. Although our present case shows that no single test system will be sufficient, we suspect that zebrafish will nonetheless prove to be useful for identifying additional regulatory elements required for *ZIC2* expression and additional HPE genes, based on the ancient requirements of forebrain development and patterning. Concurrently, mouse geneticists are working on parallel transient transgenesis approaches that may prove even more useful in translational research of human genetic variation [28]–[29]. Estimates that 4–6% of the human genome is non-coding sequence with likely regulatory function dictates that this dilemma should remain a priority for both basic scientists and clinicians.

Although we can show that the 3′UTR element is a target of mutation of likely relevant sequence changes among HPE subjects, we have yet to demonstrate the consequences of these base pair alterations. This remains a challenge for the future. Despite its similarity to neural enhancers in *Drosophila*, our element may well have unappreciated functions. Furthermore, bioinformatic analysis alone does not tell us which of the multiple sequence elements contained within the conservation block are essential, nor which elements are utilized by both species (in the same or similar way), nor which additional functions have evolved due to sequence divergence and adaption. Basic research into these questions will be essential for progress in this area.

The pattern of mutation of our *ZIC2* element in this study is entirely analogous to what is seen with the more conventional sequencing of its coding exons. The mutations are rare variants that cannot be readily extrapolated from public databases. In most cases, there will be no information on these variants in extensive public databases, or by comparisons between different diagnostic laboratories. Given this fact, we now conclude that this type of regulatory element be sequenced prospectively in all new cases. Variants of all types either from subjects or controls should be considered for functional testing whenever this is feasible. Several of our subjects were observed to have mutations in more than one HPE risk amplicon (coding and non-coding, see Table S1). This observation is likely only the tip of the iceberg. As the extent of testing of each new subject increases, so will the likelihood of “multiple hits” detected among the battery of tested genes. In the handful of cases that have been adequately examined the observed pattern tends to be of a mutation with a strong attributable risk in conjunction with a normal variant, or one with mildly abnormal function [30]–[34]. We therefore recommend that the databases of genetic variation ultimately include tests of function of both subject mutations and population variants [35]. It is becoming increasingly appreciated that even common polymorphisms can have unappreciated, yet substantial, functional effects to either buffer or enhance the biological consequences of more classical mutations.

## Supporting Information

Figure S1 **A multiple species alignment using UCSC embedded algorithm PhastCon.** The ECR#1_99 bp and ECR#2_367 bp sub-elements of the *ZIC2* 3′UTR (yellow) as identified by ECRbase (see Fig. 1) are presented as gap alignments using PhastCon. The unique variations (red) and the polymorphic changes (green) are highlighted in the sequence and numbered with reference to the last base of the coding region (c.1599). The sequence blocks identified by *cis*-Decoder retain the same color code as used in Figure 2. Note that both presumed polymorphic variants (*587G>T, green) and likely pathogenic variations (*889T>C and *954T>A, red) are present in sequence blocks that are both highly conserved by PhastCon and EvoPrinter, but also highlighted by *cis*-Decoder.
(DOC)

Figure S2 **An alignment between human and mouse sequences.** The same alignment used in Figure S1 is now simplified to compare only the human and mouse sequences. rVista allows for predictions of conserved transcription factor binding sites (TFBS in TRANSFAC databases) between two selected species (human vs. mouse). Those predicted TFBS also present in the zebrafish alignment are highlighted by green font.
(DOC)

Figure S3 **An alignment between human and zebrafish sequences.** The same alignment used in Figure S1 is now simplified to compare only the human and mouse sequences. rVista allows for predictions of conserved transcription factor binding sites (TFBS) between two selected species (human vs. zebrafish). Those predicted TFBS also present in the mouse alignment are highlighted by green font.
(DOC)

Figure S4 **Evaluation of** ***ZIC2*** **3′ UTR elements in zebrafish.** Animal pole (A–I) and lateral (J–M) views of 6 hour post-fertilization [hpf](A–C & G–I), 8 hpf (D–F), 26 hpf (J, K) and 50 hpf embryos. Embryos were injected with either pZED vector (B, E, H, J & L), pZED800ZIC2 (C, F, I, K & M) or not injected at all (A, D & G) as a secondary negative control. (A–F) Whole-mount *in situ* hybridization with *gfp* anti-RNA probe revealed punctate BM purple staining in most vector-injected embryos that is often concentrated in the dorsal organizer region. We believe this is an artifact caused by direct hybridization of the antisense *gfp* RNA probe to the sense *gfp* DNA of the vector. In support of this interpretation, whole-mount immunostaining with anti-GFP antibody (G–I) revealed an absence of GFP immunostaining in 6 hpf embryos (J–M) Overlay of DIC, GFP and RFP stacks of confocal images. Arrowheads indicate the red fluorescent signal from the internal control cardiac actin promoter of the pZED vector. No GFP fluorescence was seen in any of the 67 pZED vector-injected or the 88 pZED800ZIC2-injected animals at the two stages shown or at earlier stages (data not shown). e, eye; f, forebrain; h, hindbrain; ht, heart; m, midbrain.
(JPG)

Table S1 **Summary of clinical and molecular findings in subjects.**
(DOC)
