# Supplementary material for: Unique Alterations of an Ultraconserved Non-Coding Element in the 3′UTR of ZIC2 in Holoprosencephaly
Source: PLoS One. 2012 Jul 31;7(7):e39026. doi: 10.1371/journal.pone.0039026 (PMC3409191; doi:10.1371/journal.pone.0039026)
Supplement: Figure S1 — A multiple species alignment using UCSC embedded algorithm PhastCon. The ECR#1_99 bp and ECR#2_367 bp sub-elements of the ZIC2 3′UTR (yellow) as identified by ECRbase (see Fig. 1) are presented as gap alignments using PhastCon. The unique variations (red) and the polymorphic changes (green) are highlighted in the sequence and numbered with reference to the last base of the coding region (c.1599). The sequence blocks identified by cis-Decoder retain the same color code as used in Figure 2. Note that both presumed polymorphic variants (*587G>T, green) and likely pathogenic variations (*889T>C and *954T>A, red) are present in sequence blocks that are both highly conserved by PhastCon and EvoPrinter, but also highlighted by cis-Decoder. (DOC) [file pone.0039026.s001.doc]

**Multiple species alignment of ECR#1 [100638485 – 100638583 99bp]**

**(*578T>A) (*587G>T)**

**Human ttgta-tt-gtggta--ttgaatattgtgttc----------c-ttt-t-tat--ga-ggc-aacctgattgt-aaacttc--atgt—aactatag**

**Human ttgta-tt-gtggta--ttgaatattgtgttc----------c-ttt-t-tat--ga-ggc-aacctgattgt-aaacttc--atgt—aactatag**

**Rhesus ttgta-tt-gtggta--ttgaatattgtgttc----------c-ttt-t-tat--ga-ggc-aacctgattgt-aaacttc--atgt--aactatag**

**Mouse ttgta-tt-gtggta--ttaaatattgtgttc----------c-ttt-taaat--ga-ggc-aacttgattgt-aaacttc--atgc--gactatag**

**Dog -------------------------------------------------------------------------------------------------**

**Elephant ttgta-tt-gtggta--ttgaatattgtgttc----------c-ttt-t-tat--ga-ggc-aacttgattgt-aaacttc--atgc--aactatag**

**Opossum ttgta-tt-gtgata--ttgaatattgtgttc----------c-ttt-t-tat--ga-ggc-aacttgattgt-aaacttc--atgc--aactatag**

**Chicken ttgtactc-gtgatctcttgaatatcgtgttc----------c-ttt-c-caa--ga-ggc-gacttgattgtaaaaccgc--atctctcaccgta-**

**X. tropicalis cttga-tt-cgtccg--tttaatatgaagtttatttaattttc-ttt-t-tattaga-gga-atcctggctgt-ca-tttc--atgc--cccaaaat**

**Zebrafish ttgta-tt-atcata--atgaatattgtgatc----------c-cat-t------aa-ggc-aaactgattgt-aa---ac-taaac--aactatta**

**Human actgg-----------------------------------------------------aaaaaa-----t------------gagccgtgccaaagtc**

**Rhesus actgg---------------------------------a-------------------aaaaaa-----c------------gagccgtgccaaagtc**

**Mouse actgg-----------------------------------------------------aaaata-----t------------gagccgtgccaaagtc**

**Dog act----------------------------------ga-------------------aaaaac-----c------------gagccgtgccaaagtc**

**Elephant act----------------------------------gg-------------------aaaaaa-----c------------gaaccgtgccaaagtc**

**Opossum act-----------------------------------g-------------------gaagaa-----a------------aaaccgtgccaaagtc**

**Chicken ccc----------------------------------gg-------------------ggaaga-----a------------gtgccgtgccaaagtc**

**X. tropicalis gat---------------------gggggggggggggga-------------------caaaaa-----c------------aa---gtgccaaagtc**

**Zebrafish act---------------------------------------------------------ggaa-----a------------ga---gtgccaaagtc**

**Multiple species alignment of ECR#2 [100638645 – 100639011 367 bp]**

**Human gcttgtgaatgta-------------------t--t-tttctgttagct-g-ggttt-acatgtgatg-tttta-gtgc-ttttgcaa-gttcaattt**

**Human gcttgtgaatgta-------------------t--t-tttctgttagct-g-ggttt-acatgtgatg-tttta-gtgc-ttttgcaa-gttcaattt**

**Rhesus gcttgtgaatgta-------------------t--t-tttctgttagct-g-ggttt-acatgtgatg-tttta-gtgc-ttttgcaa-gttcaattt**

**Mouse gcttgtgaatgta------------------tt--t-tttctgttagct-g-ggttt-acatgtgatg-tttca-gtgc-ttttgcgacgttcgattt**

**Dog gcttgtgaatgta-------------------ttat-tttctgttagct-g-ggttt-acatgtgatg-tttta-gtgc-ttttgcaa-gttcaattc**

**Elephant gcttgtgaatgta-------------------t--t-tttctgttagct-g-ggttt-acatgtgccg-ttgtc-gtgc-ttttgcaa-gttcgattt**

**Opossum gcttgtgaatgta-------------------t--t-tttctgttagct-g-ggttt-acatgtgatg-tttta-gtgc-ttttgcaa-gttcaattt**

**Chicken -----------------------------------t-ccgctgttagct-g-ggtgc--cgtgtgctg-tcgta-gtgc-tttgcc---gtccgctcc**

**X. tropicalis gcttgtgaatgta-------------------c--t-tttctgttaaat-g-ggctt----tgtgatg-ttttctgtgc-ttttgcaa-gttgaattt**

**Zebrafish gcttgtgaatgta-----------------tat--c-tt--agtttgct-g-ggcttaacttgtgatg-tttt--gtgc--tttgcaa-gtttgaatt**

**(836C>T)**

**Human gttagttc--ctgta-tgaaagattgt------ggggg--------aaaaa ta------aacgt--cgtgccgttagc-t-ttt-tccgtaataaca**

**Human gttagttc--ctgta-tgaaagattgt------ggggg--------aaaaa ta------aacgt--cgtgccgttagc-t-ttt-tccgtaataaca**

**Rhesus gttagttc--ctgta-tgaaagattgt------ggggg--------aaaaa ta------aacgt--cgtgccgttagc-t-ttt-tccgtaataaca**

**Mouse gttagttc--ttgta-tgaaagattgg------ggggtgggtgggtgggag ta------aacgt--tgtgccgttagctt-ttt-tccgaaataaca**

**Dog gttagttc--ctgta-tgaaagattgt---------gg--------gggaa ta------aacgt--tgtgccgttagc---ttt-tccgtcataaca**

**Elephant gttagttt--ctgta-tgaaagattgt------ggggg--------tgggg tc------aacgt--ggtgcccttagc-ttttc-tccctaataaca**

**Opossum gttagttc--ctgta-cggaagattgt------gggga--------aaaa- ta------aacgt--tgtgccgttagc-t-ttt-tccgtaataaca**

**Chicken ctttagtc--ctggt-gggaaggttgt------gagg----------aaa- tc------agcgt--cgtgccgtaggc---ttc--ccgt--ccacc**

**X. tropicalis gttagtaa--ctgtt-tggaaaagcgc------cgagg--------caaaa ta------gccgt--tgtgcactc-----------gccccatagca**

**Zebrafish gtgaaata---tatt-tggaagattgt------ggggc--------aaa-- tt------aatgt--cgtgcggttaaa-t-at---ctgtaactaca**

**(*889T>C) (*899A>G)**

**Human c--cc-t--tccttctgtaaatacccgttaccatatttatccatttgtaattaaattatggtattaacttgctacagaggaaacaatatttat-aaag**

**Human** c--cc-t--tccttctgtaaatacccgttaccatatttatccatttgtaattaaattatggtattaacttgctacagaggaaacaatatttat-aaag

Rhesus c--cc-t--tccttctgtaaatacccgttaccatatttatccatttgtaattaaattatggtattaacttgctacagaggaaacaatatttat-aaag

Mouse c---------ccttctgtaaatatccgttgccatatttatccatttgtaattaaattatggtattaacttgctacagaggaaacaatatttat-aaag

Dog c--cc-t--tccttctgtaaatacccgttaccatatttatccatttgtaattaaattatggtattaacttgctacagaggaaacaatatttat-aaag

Elephant c--ct-t--tccttctgtaaataccctttgccatatttatccatttgtaattaaattatggtattaacttgctacagaggaaacaatatttat-aaag

Opossum c--cc-t--tccttctgtaaatatccattaccatatttatccatttgtaattaaattatggtattaacttgctacagaggaaacaatatttat-aaag

Chicken g--cc-c--tcctcctgtaaatacccgtgaccgtatttatccatttgtaattaaattatggtattaacttgctacagaggaaacggtatttat-aaag

X. tropicalis g--cc-t--tccttgtgtaaataatcgctaccatatttatccatttgtaattaaattatggtattaacttgcaacagaggaaacactatttat-aaag

**Zebrafish c--cc-t--tctttttgtaaatatcgactgccatatttatccatttgtaattaaattatggtatttacttactacagatgaaacaatatttat-aaag**

**(*954T>A) (*966A>G)**

**Human aatgtttcttaactataaatatgtacaattgtgggcataaactgtttcaga--ttttttat--------------ttgaaggttttaagtggtttgat**

**Human** aatgtttcttaactataaatatgtacaattgtgggcataaactgtttcaga--ttttttat--------------ttgaaggttttaagtggtttgat

Rhesus aatgtttcttaactataaatatgtacaattgtgggcataaactgtttcaga--ttttttat--------------ttgaaggttttaagtggtttgat

Mouse aatgtttcttaactataaatatgtacaattgtgggcataaactgtttcaga--ttttttat--------------ttgaagg-tttaagtggtttgat

Dog aatgtttcttaactataaatatgtacaattgtgggcataaactgtttcaga--ttttttat--------------ttgaaggttttaagtggtttgat

Elephant aatgtttcttaactataaatatgtacaattgtgggcataaactgttccaga--ttttttat--------------ttgaaggttttaagtggtttgat

Opossum aatgtttcttaactataaatatgtacaattgtgggcataaactgtttcaga--atttttat--------------ttgaagg-tttaagtggtttcat

Chicken aatgtttcttaactataaatatgtacaattgtgagcataaactgtttcaga-tttttttat--------------ttggagg-tttaagtggtttcat

X. tropicalis attgtttcttgactataaatatgtacatttgtgagcataaatgttttcaga--tttttttt--------------tttta--ttttatgtggtctctt

**Zebrafish aatgtttctttactataaatatgtacaataatgggc-taaacaggggcatt--tgttttgttatatgtgtttttgtttaaag-tttgagaagtgtttt**

**Human cat--ttcttgtg--a--tgtttt--gagag---taatgcatacagaaatataat-aaaatgtgttg**

Rhesus cat--ttcttgtg--a--tgtttt--gagag---taatgcatacagaaatataat-aaaacgtgttg

Mouse cat--ttcttgtg--a--tgtttt--gagag---taatgcatacagaaatataat-aaaatgtgttg

Dog cat--ttctcgtg--atatgtttt--gagag---taatgcatacagaaatataat-aaaatgtgttg

Elephant cat--ttcttgtg--atatgtttt--gagag---taatgcatacagaaatataat-aaaatgtgttg

Opossum cat--ttcttgtg--atatgtttt--aagag---taatgcatacagaaatataat-aaaatgtgttg

Chicken cct--ttctcgtg--atatgtttg--gagag---taatgcgtacagaactataat-aaaacgtgttg

X. tropicalis cat--tt-ttgtg--acatgtttt--aaaag---taatgcatacagacctctaat-aaaatgtgttg

**Zebrafish ctccattattgtg--a-------t---aaaattttactgcatac--agatataat-aaaacgtggtg**
