# Supplementary material for: Unique Alterations of an Ultraconserved Non-Coding Element in the 3′UTR of ZIC2 in Holoprosencephaly
Source: PLoS One. 2012 Jul 31;7(7):e39026. doi: 10.1371/journal.pone.0039026 (PMC3409191; doi:10.1371/journal.pone.0039026)
Supplement: Figure S2 — An alignment between human and mouse sequences. The same alignment used in Figure S1 is now simplified to compare only the human and mouse sequences. rVista allows for predictions of conserved transcription factor binding sites (TFBS in TRANSFAC databases) between two selected species (human vs. mouse). Those predicted TFBS also present in the zebrafish alignment are highlighted by green font. (DOC) [file pone.0039026.s002.doc]

**Multiple species alignment of ECR#1 [100638485 – 100638583 99bp]**

**MRF2_01 TTGTA-TT-GTGgta**

**AML1_01 T-GTGGT**

**CDP_01 A--TTGAATATTGT**

**(*578T>A) (*587G>T)**

**Human ttgta-tt-gtggta--ttgaatattgtgttc----------c-ttt-t-tat--ga-ggc-aacctgattgt-aaacttc--atgt--aactatag**

**||||| || |||||| ||:|||||||||||| | ||| | :|| || ||| |||:||||||| ||||||| |||: :|||||||**

**Mouse ttgta-tt-gtggta--ttaaatattgtgttc----------c-ttt-taaat--ga-ggc-aacttgattgt-aaacttc--atgc--gactatag**

**CEBP_C t------------gAGCCGTGCCAAAGTC**

**E2F_Q6 gccgtgccaaag**

**E2F_Q4 ccgtgccaaag**

**E2F1_Q4 cgtgccaaa**

**SP3_Q3 ccgtgccaaagtc**

**Human actgg-----------------------------------------------------aaaaaa-----t------------gagccgtgccaaagtc**

**||||| ||||:| | ||||||||||||||||**

**Mouse actgg-----------------------------------------------------aaaata-----t------------gagccgtgccaaagtc**

**Multiple species alignment of ECR#2 [100638645 – 100639011 367 bp]**

**PITX2_Q2 ct-g-ggttt-aca**

**MYCMAX_02 tt-acatgtgatg**

**TFE_Q6 acatgtga**

**Human gcttgtgaatgta-------------------t--t-tttctgttagct-g-ggttt-acatgtgatg-tttta-gtgc-ttttgcaa-gttcaattt**

**||||||||||||| | | |||||||||||| | ||||| |||||||||| |||:| |||| ||||||:| ||||:||||**

**Mouse gcttgtgaatgta------------------tt--t-tttctgttagct-g-ggttt-acatgtgatg-tttca-gtgc-ttttgcgacgttcgattt**

**PAX_Q6 gttagttc—ctg**

**CREB_Q4 cgtgccgttagc**

**VMYB_01,VMYB_02 tgccgttagc**

**TBX5_01 aataacacctt**

**(*836C>T)**

**Human gttagttc--ctgta-tgaaagattgt------ggggg--------aaaaa ta------aacgt--cgtgccgttagc-t-ttt-tccgtaataaca**

**|||||||| :|||| ||||||||||: ||||: :::|: || ||||| :||||||||||| | ||| ||||:|||||||**

**Mouse gttagttc--ttgta-tgaaagattgg------ggggtgggtgggtgggag ta------aacgt--tgtgccgttagctt-ttt-tccgaaataaca**

**RFX1_02 ccgttaccatatttatcc**

**CDPCR3_01 ccatatttatccatt**

**CDX_Q5 catatttatccatttgta**

**TBP_01 tatttatc**

**POU1F1_Q6 atttatccat**

**BRN2_01 tccatttgtaattaaa**

**CDX2_Q5 gtaattaaattat**

**LHX3_01 gtaattaaat**

**NKX25L_01 aattatg**

**MYB_Q6 attaacttgc**

**NFAT_Q6 cagaggaaacaa**

**XFD3_01 agaggaaAcaatat**

**FOXJ2_02 gaaacaatatttat**

**SOX5_01 gaaacaatat**

**XFD1_01,XFD2_01 acaatatttat-aaa**

**TBP_01 tatttat-a**

**CDXA_02 atttat-a**

**(*889T>C) (*899A>G)**

**Human c--cc-t--tccttctgtaaatacccgttaccatatttatccatttgtaattaaattatggtattaacttgctacagaggaaacaatatttat-aaag**

**| |||||||||||||:|||||:||||||||||||||||||||||||||||||||||||||||||||||||||||||||||||||| ||||**

**Mouse c---------ccttctgtaaatatccgttgccatatttatccatttgtaattaaattatggtattaacttgctacagaggaaacaatatttat-aaag**

**RFX1_01 atgtttcttaactataa**

**MEF2_02 ttcttaactataaatatgtaca**

**FREACT7_01 taactataaatatgta**

**XFD2_01 actataaatatgta**

**TBP_01 tataaata**

**CDXA_02 tataaat**

**SRF_01 atgtacaattgtgggcat**

**CBF_02 acaattgtgggcataa**

**AREB6_04 ctgtttcag**

**FOXD3_01 ga--ttttttattt**

**HFH1_01 ga—TTTTTTATT**

**NKX22_01, NKX25_01 ttaagtggtt**

**SREBP1_02 gtggtttgatc**

**GATA3_03 tttgatcatt**

**(*954T>A) (*966A>G)**

**Human aatgtttcttaactataaatatgtacaattgtgggcataaactgtttcaga--ttttttat--------------ttgaaggttttaagtggtttgat**

**||||||||||||||||||||||||||||||||||||||||||||||||||| |||||||| ||||||| |||||||||||||||**

**Mouse aatgtttcttaactataaatatgtacaattgtgggcataaactgtttcaga--ttttttat--------------ttgaagg-tttaagtggtttgat**

**STAT5A_04 cat—ttctt**

**CHOP_01 gag---taatgcatac**

**PIT1_Q6 aatgcatacagaaatata**

**PAX3_01 t-aaaatgtgttga**

**Human cat--ttcttgtg--a--tgtttt--gagag---taatgcatacagaaatataat-aaaatgtgttg**

**||| |||||||| | |||||| ||||| ||||||||||||||||||||| |||||||||||**

**Mouse cat--ttcttgtg--a--tgtttt--gagag---taatgcatacagaaatataat-aaaatgtgttg**
