# Supplementary material for: Unique Alterations of an Ultraconserved Non-Coding Element in the 3′UTR of ZIC2 in Holoprosencephaly
Source: PLoS One. 2012 Jul 31;7(7):e39026. doi: 10.1371/journal.pone.0039026 (PMC3409191; doi:10.1371/journal.pone.0039026)
Supplement: Figure S3 — An alignment between human and zebrafish sequences. The same alignment used in Figure S1 is now simplified to compare only the human and mouse sequences. rVista allows for predictions of conserved transcription factor binding sites (TFBS) between two selected species (human vs. zebrafish). Those predicted TFBS also present in the mouse alignment are highlighted by green font. (DOC) [file pone.0039026.s003.doc]

**Multiple species alignment of ECR#1 [100638485 – 100638583 99bp]**

**MRF2 tgaatattgtgttc**

**CDP a--ttgaatattgt**

**OCT1 gta--ttgaatattgtg**

**(*578T>A) (*587G>T)**

**Human ttgta-tt-gtggta--ttgaatattgtgttc----------c-ttt-t-tat--ga-ggc-aacctgattgt-aaacttc—atgt--aactatag**

**||||| || :|::|| :|||||||||||:|| | ::| | :| ||| ||:|||||||| || :| :::: ||||||::**

**Zebrafish ttgta-tt-atcata--atgaatattgtgatc----------c-cat-t------aa-ggc-aaactgattgt-aa---ac-taaac—aactatta**

**Human actgg-----------------------------------------------------aaaaaa-----t------------gagccgtgccaaagtc**

**||| ::|| : || |||||||||||**

**Zebrafish act---------------------------------------------------------ggaa-----a------------ga---gtgccaaagtc**

**Multiple species alignment of ECR#2 [100638645 – 100639011 367 bp]**

**Human gcttgtgaatgta-------------------t--t-tttctgttagct-g-ggttt-acatgtgatg-tttta-gtgc-ttttgcaa-gttcaattt**

**||||||||||||| | : || :|||:||| | ||:|| ||:||||||| |||| |||| ||||||| |||::||||**

**Zebrafish gcttgtgaatgta-----------------tat--c-tt--agtttgct-g-ggcttaacttgtgatg-tttt--gtgc--tttgcaa-gtttgaatt**

**(*836C>T)**

**Human gttagttc--ctgta-tgaaagattgt------ggggg--------aaaaa ta------aacgt--cgtgccgttagc-t-ttt-tccgtaataaca**

**||:|::|: |:|: ||:|||||||| ||||: ||| |: ||:|| |||||:||||:: | :| |:||||::|||**

**Zebrafish gtgaaata---tatt-tggaagattgt------ggggc--------aaa-- tt------aatgt--cgtgcggttaaa-t-at---ctgtaactaca**

**CDPCR3_01 ccatatttatccatt**

**CDX_Q5 catatttatccatttgta**

**TBP_01 tatttatc**

**POUF1_Q6 atttatccat**

**BRN2_01 tccatttgtaattaaa**

**CDX2_Q5 tgtaattaaattat**

**LHX3_01 gtaattaaat**

**NKX25L_01 aattatg**

**FOXJ2_02 gaaacaatatttat**

**SOX5_01 gaaacaatat**

**XFD1_01,XFD2_01 acaatatttat-aaa**

**TBP_01 tatttat-a**

**CDXA_02 atttat-a**

**(*889T>C) (*899A>G)**

**Human c--cc-t--tccttctgtaaatacccgttaccatatttatccatttgtaattaaattatggtattaacttgctacagaggaaacaatatttat-aaag**

**| || | |||||:||||||||:|:::|:||||||||||||||||||||||||||||||||||||||||:|||||||:|||||||||||||| ||||**

**Zebrafish c--cc-t--tctttttgtaaatatcgactgccatatttatccatttgtaattaaattatggtatttacttactacagatgaaacaatatttat-aaag**

**CDXA_02 tataaat**

**TBP_01 tataaata**

**XFD2_01 actataaatatgta**

**FREACT7_01 taactataaatatgta**

**MEF2_02 ttcttaactataaatatgtaca**

**(*954T>A) (*966A>G)**

**Human aatgtttcttaactataaatatgtacaattgtgggcataaactgtttcaga--ttttttat--------------ttgaaggttttaagtggtttgat**

**||||||||||||||||||||||||||||||:||||| |||||:|:::||:| |:||||:| ||:||:| |||:||::||:|::|**

**Zebrafish aatgtttctttactataaatatgtacaataatgggc-taaacaggggcatt--tgttttgttatatgtgtttttgtttaaag-tttgagaagtgtttt**

**Human cat--ttcttgtg--a--tgtttt--gagag---taatgcatacagaaatataat-aaaatgtgttg**

**|:: ||:||||| | | |:|: ||:||||||| |:||||||| ||||:||||||**

**Zebrafish ctccattattgtg--a-------t---aaaattttactgcatac--agatataat-aaaacgtggtg**
