# Supplementary figures and images for: Unique Alterations of an Ultraconserved Non-Coding Element in the 3′UTR of ZIC2 in Holoprosencephaly
Source: PLoS One. 2012 Jul 31;7(7):e39026. doi: 10.1371/journal.pone.0039026 (PMC3409191; doi:10.1371/journal.pone.0039026)

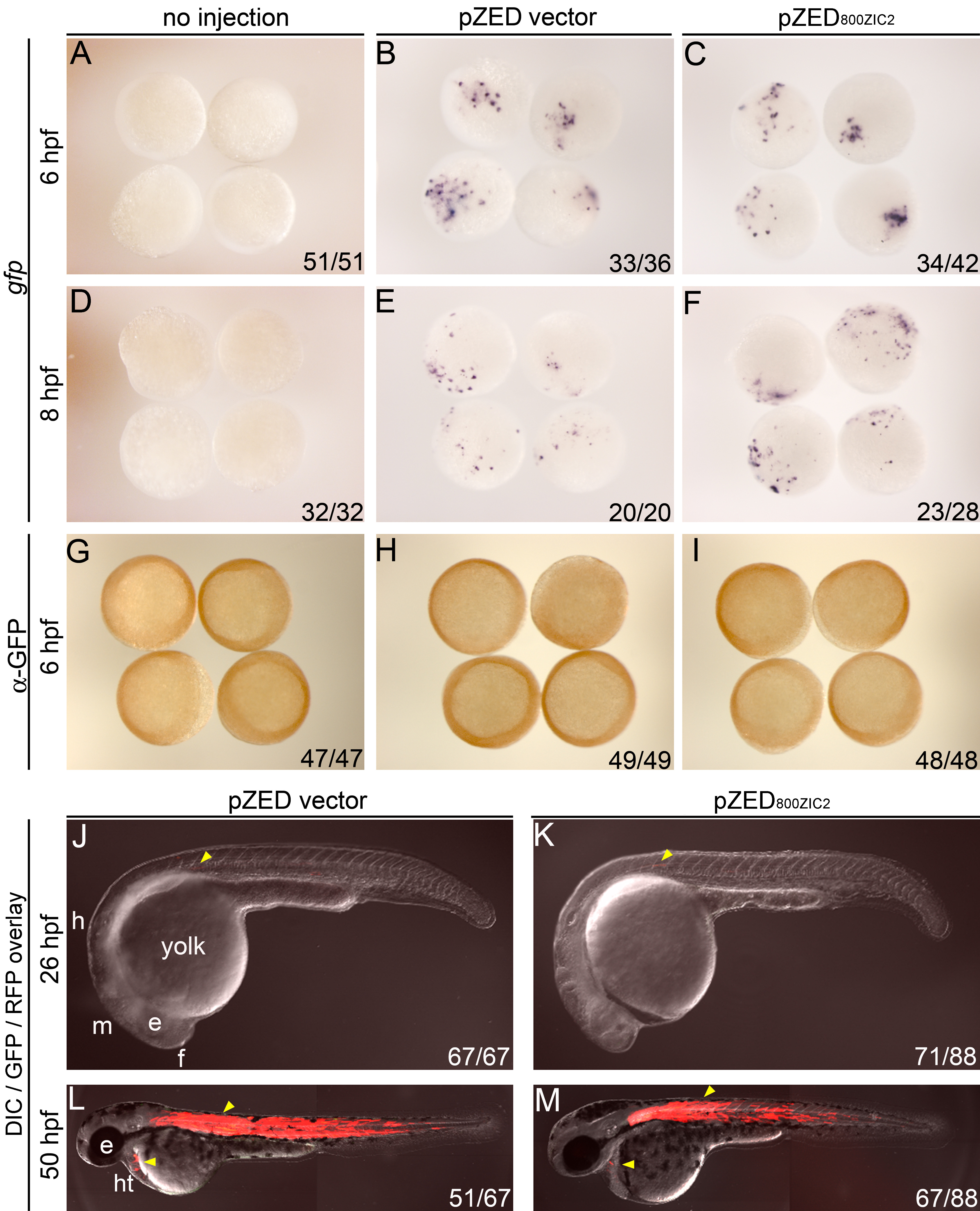

Supplement: Figure S4 — Evaluation of ZIC2 3′ UTR elements in zebrafish. Animal pole (A–I) and lateral (J–M) views of 6 hour post-fertilization [hpf](A–C & G–I), 8 hpf (D–F), 26 hpf (J, K) and 50 hpf embryos. Embryos were injected with either pZED vector (B, E, H, J & L), pZED800ZIC2 (C, F, I, K & M) or not injected at all (A, D & G) as a secondary negative control. (A–F) Whole-mount in situ hybridization with gfp anti-RNA probe revealed punctate BM purple staining in most vector-injected embryos that is often concentrated in the dorsal organizer region. We believe this is an artifact caused by direct hybridization of the antisense gfp RNA probe to the sense gfp DNA of the vector. In support of this interpretation, whole-mount immunostaining with anti-GFP antibody (G–I) revealed an absence of GFP immunostaining in 6 hpf embryos (J–M) Overlay of DIC, GFP and RFP stacks of confocal images. Arrowheads indicate the red fluorescent signal from the internal control cardiac actin promoter of the pZED vector. No GFP fluorescence was seen in any of the 67 pZED vector-injected or the 88 pZED800ZIC2-injected animals at the two stages shown or at earlier stages (data not shown). e, eye; f, forebrain; h, hindbrain; ht, heart; m, midbrain. (JPG) [file pone.0039026.s004.jpg]
