# Supplementary material for: Unique Alterations of an Ultraconserved Non-Coding Element in the 3′UTR of ZIC2 in Holoprosencephaly
Source: PLoS One. 2012 Jul 31;7(7):e39026. doi: 10.1371/journal.pone.0039026 (PMC3409191; doi:10.1371/journal.pone.0039026)
Supplement: Table S1 — Summary of clinical and molecular findings in subjects. (DOC) [file pone.0039026.s005.doc]

| Variant | Subject | HPE Type | Enhancer Inheritance | *SHH* | *ZIC2* | *SIX3* | *TGIF* |
| --- | --- | --- | --- | --- | --- | --- | --- |
| c.1599*456G>A | rs13542 dbSNP | N.A. | N.A. | N.A. | N.A. | N.A. | N.A. |
| c.1599*578T>A | LCL1349 | Microform | *De novo* | c.419A>C  p.His140Pro (Mat) | - | - | - |
| c.1599*587G>T | FB9622, LCL7282, LCL6386 | N.A.  N.A.  N.A. | N.A.  N.A.  N.A. | -  -  - | -  -  - | - | c.487C>T  p.Pro163Ser^a^  -  - |
| c.1599*836C>T | Brz-2172 | Semilobar, normal face | Mat neg  Father not available | - | c.1215dupC  p.Ser406Glnfs*11^c^ | - |  |
| c.1599*889T>C | AM6632 | Alobar | Parents not available | - | - | - | c.420A>G  p.Pro140Pro^a^  c.488C>T  p.Pro163Leu^a^ |
| c.1599*899A>G | LCL301  LCL7897^b^ | Microform, cleft lip and hypotelorism | Pat positive  Pat positive | c.72C>A  p.Cys24* (Pat)  c.72C>A  p.Cys24* (Pat) | c.1059C>T  p.His353His^a^ | - | - |
| c.1599*954T>A | Brz-37 | Semilobar, microcephaly, right cleft lip and palate | *De novo* | - | - | - | - |
| c.1599*966A>G | LCL7828 | Semilobar | Mat positive  Father not available | - | c.1059C>T  p.His353His^a^ | - | c.289A>G  p.Met97Val (not present in mother, father not available) |

^a^ common variant in healthy individuals.

^b^ affected sibling of proband LCL301 (both have CLIA confirmed paternal inheritance of SHH p.Cys24*).

^c^ *in cis* with enhancer variant by co-amplification and sequencing.
